# Artificial intelligence in the surgical management for colorectal cancer: current applications and future directions

**DOI:** 10.3389/fonc.2026.1947280

**Published:** 2026-09-25

**Authors:** Waiian Cheang, Zihan Han, Jianzheng Jie

**Affiliations:** 1 Department of Colorectal Surgery, China-Japan Friendship Hospital, Beijing, China; 2 China-Japan Friendship Hospital (Institute of Clinical Medical Sciences), Chinese Academy of Medical Sciences & Peking Union Medical College, Beijing, China

**Keywords:** colorectal cancer, artificial intelligence, clinical applications, clinical decision support, intraoperative navigation

## Abstract

Artificial intelligence (AI) is being increasingly investigated across colorectal cancer (CRC) management, with the potential to improve diagnostic accuracy, support surgical decision-making, and refine prognostic stratification. This review focuses on AI applications within the surgical treatment continuum for CRC, covering endoscopic screening and diagnosis, radiological staging, intraoperative navigation, postoperative complication prediction and prognostic risk stratification. From a surgeon-centered perspective, we critically analyze the gap between model performance and real-world clinical utility, as well as key translational barriers. We believe this review provides a foundation for clinicians and researchers seeking to understand the current state and future potential of AI in CRC surgical care.

## 1 Introduction

Colorectal cancer (CRC) ranks as the third most common cancer and the second leading cause of cancer-related deaths worldwide (1), with the global burden projected to reach 3.2 million annually by 2040 (2). CRC surgical management encompasses a continuum, from screening, diagnosis and staging, through surgical intervention, to postoperative surveillance and prognostication. Precision is paramount at each step, timely screening enables early diagnosis, accurate staging guides treatment strategy, high-quality surgery minimizes complications and recurrence, and reliable risk stratification guide adjuvant therapy or corresponding medical treatment decisions. Despite advances in systemic therapies, surgery remains the cornerstone of curative treatment, and its technical quality directly determines both short-term morbidity and long-term oncological outcomes. However, several persistent challenges undermine this continuum infect the management pathway. Traditional endoscopic lesion characterization remains subjective, surgical skills are heavily experiment-dependent, and current prognostic tools lack sufficient accuracy for personalized decision-making.

Artificial intelligence (AI) has emerged as a promising solution to many of these challenges, with current applications spanning AI-assisted diagnosis, intraoperative surgical application, and postoperative outcome prediction. Despite favorable performance metrics, the clinical translational gap between model development and meaningful clinical impact remains substantial. For a surgeon’s standpoint, AI assistance should genuinely improve clinical decision-making rather than merely demonstrate statistical superiority. Also, AI must be seamlessly integrated into CRC management pathway with efficiency enhancement. And real-time surgical assistance with prospectivity and not compromising clinical efficiency.

This review is structured along the surgical workflow of CRC management, encompassing preoperative diagnosis and decision-making, intraoperative navigation, and postoperative management. For this narrative review, we searched PubMed, Web of Science, and the Cochrane Library (1^st^ January 2020–1^st^ June 2026) using terms related to colorectal cancer, AI, and surgical applications, and included original studies on diagnosis, navigation, complication prediction, or prognosis. Additional landmark studies were identified through hand-searching. We summarize AI applications at each stage and critically analyze the translational challenges from a surgeon-centered perspective, providing a foundation for future interdisciplinary research.

## 2 Screening and diagnosis under colonoscopy: application of AI in colonoscopy

AI-assisted colonoscopy systems address operator-dependent variability of traditional colonoscopy by standardizing visual inspection. Current research focuses on three main areas: lesion detection (CADe), lesion characterization (CADx), and prediction of submucosal invasion depth to determine endoscopic resectability.

### 2.1 Lesion detection

CADe systems assist endoscopists in identifying polyps in real time. Landmark trial conducted by Wang et al. demonstrated that CADe significantly increased adenoma detection rate (ADR) from 20.3% to 29.1% (3). A subsequent tandem study confirmed that CADe reduced overall adenoma miss rate (AMR) from 40.0% to 13.9%, particularly for diminutive adenomas(≤5mm) (4). Other critical randomized controlled trials have consistently demonstrated that CADe significantly improves efficacy of colonoscopy, with ADR ranging from 44.5% to 53.3% in CADe cohorts, and AMR ranging from 15.5% to 20.1% in CADe cohorts compared with 31.3% to 32.4% in control cohorts (5–8).

Two large-scale meta-analysis confirmed that CADe significantly improved ADR by approximately 20% and reduced AMR by more than half, while also revealing considerable heterogeneity across study designs (9, 10). This heterogeneity was further illustrated by Nayar et al., who found in a U.S. single-center propensity-matched study that the CADe-driven gains were predominantly driven by diminutive (≤5 mm) and hyperplastic polyps of low clinical significance, raising doubts about its net clinical benefit in routine practice (11). These observations underscore the need for a standardized assessment framework to better evaluate CADe efficacy across diverse clinical contexts.

Apart from metrics on AMR or ADR, evidence regarding sessile serrated lesions (SSLs) detection remains inconclusive. Meta-analysis have yielded conflicting results, with one reporting a modest but significant improvement (RR 1.21; 95% CI 1.04–1.42) and another finding no significant benefit (RR 1.10; 95% CI 0.93–1.30) (9, 10). Two prospective trials evaluating the same GI Genius system showed divergent outcomes same as above: the single-center COLO-GENIUS trial found no significant improvement in SSL detection (12), whereas the multi-center COLO-DETECT trial reported a significant increase (adjusted OR 1.46) (13). Notably, the EAGLE trial, employing a cloud-based CADe system trained on an enriched multi-center dataset, demonstrated a more than three-fold increase in SSL detection rate (from 0.03 to 0.08 per colonoscopy) (14), suggesting enriched training on multi-center datasets may improve detection performance.

Technically, CADe standardizes scan quality and highlights suspicious areas, reducing operator-dependent variability, but provides no histologic information. Practically, CADe offers patients fewer missed lesions and theoretically reduced interval cancer risk, but long-term evidence is lacking. For surgeons, CADe has limited direct impact on surgical decision-making.

### 2.2 Lesion characterization

Compare with CADe, computer-aided diagnosis (CADx) tackles lesion characterization. By performing optical biopsy during colonoscopy, CADx predicts polyp histology in real time, informing whether to remove, leave, or refer for surgery. Yet its performance remains inconsistent across studies.

Several studies have reported notable performance of CADx, with some achieving accuracy metric comparable to or higher than unassisted endoscopists’ accuracy metrics (15–19). But results are not universally reproducible. The POLAR system reported by Houwen et al. showed high accuracy of 79% and sensitivity of 89% but poor specificity of 37.8% and minimal SSL detection capability (sensitivity 17.1%) in a prospective multi-center study (20). Similarly, the CAD EYE system conducted by Li et al. demonstrated inconsistent performance across studies, with one trial reporting accuracy inferior to experts (71.6% vs. 75.2%) in a prospective multi-center study (21), while another reported by De Lange et al. in another prospective multi-center setting found comparable overall accuracy (81% vs. 86%) but significantly lower sensitivity and frequent SSL misclassification (22 of 23) (22). These inconsistencies are corroborated by two meta-analysis, which concluded that CADx does not provide incremental clinical benefit over endoscopists and fails to meet the performance thresholds required for optical biopsy strategies (23, 24).

From a surgical perspective, CADx is more relevant than CADe, as its ability to predict deep invasion guides surgical referral. Although several CADe/CADx systems have received regulatory approval such as GI Genius (FDA-cleared in the US; CE-marked in Europe), CAD EYE (CE-marked in Europe), EndoScreener (NMPA-approved in China; FDA-cleared in the US; CE-marked in Europe), ENDOANGEL (NMPA-approved in China) and EndoBRAIN-Plus (PMDA-approved in Japan), their real-world adoption remains limited. This is largely due to persistent challenges in ensuring consistent performance across lesion types, particularly SSLs, along with frequent SSL misclassification and inconsistent sensitivity for invasive cancer. CADx performance in meeting the stringent negative predictive value (NPV) thresholds (≥90%) required for resect-and-discard strategies has been inconsistent across studies, directly reflecting these challenges. Until prospective validation demonstrates consistently high performance across all lesion types, CADx remains promising but clinically unproven (25).

### 2.3 Invasion depth prediction

Accurate prediction of submucosal invasion depth is the most critical task in colonoscopy, dictating endoscopic resection vs. surgical referral. For early T1 CRC, distinguishing T1a (<1000 μm) from T1b (≥1000 μm) is key, as deeper invasion increases lymph node metastasis risk. Misclassification has serious consequences: overestimation leads to unnecessary radical surgery and morbidity, while underestimation risks incomplete resection and disease progression.

Several retrospective studies have demonstrated the feasibility of AI-based invasion depth assessment, however, the reported accuracies and sensitivities vary considerably across studies for distinguishing superficial from invasive lesions. (**Table 1**) (26–31). Notably, these values are derived from different retrospective studies with heterogeneous datasets. They have also illustrated that AI-assisted endoscopy achieved performance comparable to or higher than that of endoscopists, confirming its potential to serve as a valuable assistive tool. However, there is no standardized framework for direct comparison across such heterogeneous studies, and nearly all of these studies share critical shortcomings that they are retrospective, single-center, and based on static image analysis without real-time deployment, raising concerns about their generalizability to clinical practice. These are also the major limitations of current research on AI applications in medicine.

**Table 1:** Current studies on AI-assisted colonoscopy predicting invasive depth of lesions.

Author/year	Type of study	AI model	Classification task	Imaging modality	External validation	External/internal evaluation metrics			
						Accuracy	Sensitivity	Specificity	AUC
(26)	Retrospective/multi-center	DL	Tis/T1a vs. T1b	WLE	1 Center (78 images)	84%	81%	87%	0.888
(27)	Retrospective/single-center	DL	Suitable for ER vs. unsuitable (1000μm)	WLE	—	(Internal validation metrics)			
						91.1%	91.2%	91.0%	0.970
(28)	Retrospective/multi-center	DL	Suitable for ER vs. unsuitable (1000μm)	WLE	—	(Internal validation metrics)			
						90.3%	96.7%	75.0%	0.913
(29)	Retrospective/multi-center	DL	Suitable for ER vs. unsuitable (1000μm)	WLE+IEE	2 Centers (4,528 image pairs)	88.11%	92.45%	85.56%	0.956
(30)	Retrospective/multi-center	DL	Tis/T1a vs. T1b	WLE	10 Centers (1,392 images)	87.3%	59.8%	94.4%	0.851
(31)	Retrospective +temporal validation/multi-center	DL	Suitable for ER vs. unsuitable (1000μm)	WLE + NBI + chromoendoscopy	2 Centers (349 patients)	74.4% (Validation)	87.2% (Validation) 75.7% (Temporal)	35.7% (Validation)	0.758 (Validation)

DL, Deep Learning; MS, Modified Sano classification; WLE, White Light Endoscopy; NBI, Narrow-Band Imaging; BLI, Blue Laser Imaging, IEE, Image-enhanced endoscopy; EC-NBI, Endocytoscopy with NBI ultra-magnification.

In contrast, Gong and Bang developed an edge-computing-based deep learning (DL) model for real-time histological classification of colorectal lesions into four categories (advanced cancer, early cancer/high-grade dysplasia, tubular adenoma, and non-neoplasm) in a retrospective study, achieving 83.5% accuracy for four-category classification and 94.6% for neoplasm versus non-neoplasm classification, with an inference speed of only 2–3 milliseconds per frame (32). This model has been externally validated on an independent multi-center dataset, showing equivalent performance, which has supported its transportability to the evaluated external dataset. Another distinct approach is being prospectively evaluated by the CLASSICA study led by Moynihan A et al., which aims to validate a real-time AI system that analyzes indocyanine green fluorescence perfusion patterns to classify rectal lesions as benign or malignant during transanal endoscopic surgery, representing a prospective multi-center effort to establish an alternative AI-guided tissue characterization method (33).

As the gold standard for CRC, colonoscopy critically determines the treatment direction. CADe has advanced considerably, but AI-assisted lesion characterization and invasion depth prediction still require improvement in efficacy and generalizability, constrained by technical heterogeneity, small sample-sizes, and the difficulty of conducting large-scale trials. The clinical priority should be accurately identifying lesions that require surgery, rather than optimizing metrics like ADR.

From a surgical perspective, meaningful AI integration extends beyond diagnostic accuracy. When an endoscopist encounters a lesion, the critical questions are whether it requires surgery, what type of resection is appropriate, and whether neoadjuvant therapy is indicated. Practical questions that accuracy metrics alone cannot answer. Clinically useful AI must deliver real-time, interpretable predictions that integrate seamlessly into workflows without prolonging procedures; a black-box system offering unexplained “high risk” alerts will not inspire trust or support shared decision-making, particularly when misclassification carries grave consequences. The final responsibility rests with the clinician, and AI should augment, not replace, human judgment.

For invasion depth prediction in particular, although T1a versus T1b differentiation has achieved approximately 90% accuracy, most studies rely on static images without prospective validation. The consequences of AI false negatives have received little attention. A missed invasive cancer or misclassified high-risk lesion could delay treatment and compromise curative outcomes. Future research should prioritize prospective validation in real-world settings, with emphasis on interpretability, workflow integration, and clinically meaningful endpoints such as accurate triage to surgery or endoscopy.

For the limitations mentioned above, they are not unique to CRC, similar issues have been highlighted across the broader field of AI-assisted endoscopy. A comprehensive review proposed that addressing these challenges requires standardized annotation, multi-center collaboration, and differentiated deployment strategies tailored to clinical contexts and endoscopist experience (34).

## 3 Radiological diagnosis: AI in radiological imaging for CRC staging

Once a lesion is biopsied and pathologically confirmed as malignant, radiological examination is required for clinical staging to guide subsequent treatment decisions. Computed tomography (CT) is the mainstay for preoperative staging and surveillance of CRC, while magnetic resonance imaging (MRI) is the reference standard for local staging of rectal cancer due to its superior soft-tissue contrast. AI has unlocked the ability to extract subtle, clinically relevant features from these imaging modalities, offering the potential to refine TNM staging and guide personalized treatment.

### 3.1 Primary tumor radiomic diagnosis

Yao et al. developed and validated a DL model for CRC detection in contrast-enhanced CT without bowel preparation in a retrospective large multi-center study, their model achieved excellent diagnostic performance on area under the curve(AUC) of 0.957-0.994 and significantly improved radiologists’ detection accuracy from 86.0% to 93.4%, demonstrating its potential clinical value of AI-assisted CRC detection (35). Besides, for CT colonography with bowel preparation, Endo et al. explored a technical innovation by integrating supine and prone positions to enhance detection sensitivity in a retrospective multi-center study. Their model achieved a sensitivity of 81.5% for lesions ≥6 mm in internal validation, and dual-position integration significantly improved sensitivity, from 70.6% to 83.5% for lesions ≥6 mm, highlighting the importance of leveraging standard clinical protocols to optimize AI performance (36). However, their external validation dataset lacked negative cases, making it impossible to assess the model’s specificity. And this study used sensitivity as the sole endpoint instead of validating the AI-assisted radiologist performance.

Conventional CT-based primary tumor diagnostic models’ discrimination remains limited partly due to its heavy reliance on large, well-annotated datasets. To overcome this challenge, Yang et al. proposed CRCFound, a self-supervised foundation model pretrained on >5,000 unlabeled CT images in a retrospective single-center study, achieving the area under the receiver operating characteristic curves (AUROCs) of 0.910, 0.853, and 0.940 for T, N, and M staging (37). However, relying solely on CT images for prognosis may be insufficient, as tumor biology often requires integration elements. Lately, Zhang et al. developed a fully automated 3D multimodal DL model using ^18^*F*-FDG PET/CT with a cross-multi-head attention mechanism in a retrospective multi-center study to differentiate early (T1-T2) from advanced (T3-T4) T-stage in CRC, achieving AUC of 0.869 and 0.814 in internal and external validation, respectively, achieving higher AUC than radiologists’ (AUC 0.627) (38).

For rectal cancer, AI research has centered on MRI about the clinically crucial distinction between T2 and T3 disease, as it demarcates the boundary between surgery-first and neoadjuvant therapy. Sun et al. found that radiomic features extracted from T2-weighted images could effectively distinguish T1–2 from T3–4 rectal cancer in a retrospective single-center study, with the predictive model achieving an AUC of 0.852 (39). Wu et al. constructed an automatic T-staging diagnostic platform for rectal cancer using Faster R-CNN on MRI images in a retrospective single-center study. In internal validation, the platform achieved overall AUCs of 0.99, 0.98, and 0.97 in the horizontal, coronal, and sagittal planes, respectively, with per-stage AUCs reaching 1.00 for each T-stages in the horizontal plane, demonstrating diagnostic performance comparable to that of senior radiologists. However, this perfect performance may be attributable to the lack of external validation and potential overfitting (40). For patients receiving neoadjuvant therapy, Nie et al. constructed a radiomic model integrating clinical and radiomic features to predict the post-treatment pathological T stage (ypT0–2 vs. ypT3-4) using pre-treatment MRI in a retrospective single-center study, achieving an AUC of 0.932 in the test cohort (41). Nonetheless, this model relied on pre-treatment MRI images, whereas post-treatment fibrosis and residual tumor are difficult to distinguish on imaging, potentially biasing the model’s assessment of true residual tumor extent.

Notably, the reported performance metrics are derived from different studies with distinct features, and direct cross-study comparisons of these values are difficult to interpret.

Technically, most of the studies faced the scarcity of annotated data, Zhang et al. proposed a self-supervised learning method named LoG-staging in a retrospective single-center study, it achieved higher accuracy metric to human radiologists’ in distinguishing T2 from T3 stages (42). However, 2D conversion sacrifices inter-slice spatial information essential for infiltration depth assessment. Class imbalance (T3 predominance, T1/T4 rarity) and the inherent T2/T3 distinction challenge further limit model performance, leading most studies to adopt simplified T1–2 vs. T3–4 dichotomies.

Practically, translating AI-assisted T-staging into practice is hindered by cascading uncertainties. First, the reliability of AI in distinguishing T2 from T3 remains questionable. Inherent radiological overlap and class imbalance (T3 predominance, T1/T4 rarity) undermine model discriminability, particularly for clinically critical but rare stages. Second, the robustness of current evidence is insufficient. Most models lack external validation and are trained on static pre-treatment images, ignoring post-therapy changes.

In addition, the high AUCs reported in many of these studies, often exceeding 0.90 in internal validation, reflect technical performance on curated, often single-center datasets rather than clinical readiness. Such metrics do not account for crossinstitutional variability in imaging protocols, patient populations, or the inherent difficulty of T2/T3 distinction. They also fail to address interpretability, confidence calibration, and workflow integration, all of which are prerequisites for surgeon trust and clinical adoption. Prospective validations against clinically meaningful endpoints, rather than statistical optimization alone, remains the essential next step.

These translational challenges are not unique to CRC imaging. Similar limitations have been described in AI-based hepatocellular carcinoma (HCC) imaging, where models trained on single-center data often underperform on different scanners or populations. Yet, despite these similar challenges, HCC imaging AI has produced commercially certified tools (such as IQQA-Liver) in routine use, whereas most CRC imaging AI models mostly remain research prototypes. It likely reflects several underlying reasons. First, the liver has stable anatomy, making AI tasks like volumetry more standardized, whereas the colorectum is mobile and heterogeneous, posing greater challenges. Second, HCC treatment relies heavily on imaging-based assessment, creating clear demand for AI, but CRC treatment is primarily pathology-driven, reducing urgency for imaging AI. Third, HCC follows relatively standardized imaging protocols while CRC protocols vary widely across centers, complicating validation and approval. These factors collectively contribute to slower progress of CRC imaging AI in clinical translation.

To address this disparity, concerted efforts toward prospective multi-center validation, protocol standardization, and clinically integrated trial design are required. The HCC experience offers several methodological insights for accelerating CRC imaging AI translation, including prospective multi-center validation with standardized imaging protocols, the use of domain adaptation and federated learning to improve model robustness across diverse clinical settings, and the implementation of visual explanation frameworks to support clinician understanding of AI decisions. HCC imaging AI has also actively integrated interpretability frameworks such as GradCAM and SHAP to visualize decision basis and enhance clinical trust—strategies that directly address the blackbox concern and offer actionable blueprints for CRC imaging AI translation (43).

### 3.2 Lymph node metastasis radiomic diagnosis

For the diagnosis of lymph node metastasis (LNM), conventional imaging assessment relies on size and morphology with limited accuracy (44). AI-based models have been explored to improve LNM prediction, yet a fundamental challenge persists. The inability to precisely co-register individual lymph nodes on imaging with their post-surgical pathological counterparts limits most models to patient-level rather than node-level prediction. To address this, Xia et al. developed a weakly supervised learning model named WISDOM, integrating T2-weighted imaging, apparent diffusion coefficient maps, and lymph node size features to identify malignant nodes and generate heatmaps. The model was validated across three centers in a retrospective study, achieving an AUC of 0.81 for predicting LNM and significantly improving radiologist diagnostic performance (45).

Recent studies have also explored diverse technical pathways to address this challenge. Several researchers focused on morphological and textural features of lymph nodes. Sun et al. built a multi-phase CT radiomics model to differentiate benign from metastatic enlarged nodes (≥10 mm) via textural features across three phases in a retrospective single-center study, achieving an AUC of 0.839 and a specificity of 89.1% (46). Li et al. identified morphological features (necrosis, irregular border, heterogeneous enhancement) to predict LNM in T1-stage cancer in a retrospective multi-center study, achieving a C-index of 0.89 and a specificity of 95% (47). Zhang et al. targeted the No. 253 lymph nodes in left-sided CRC in a retrospective single-center study, integrating clinical features such as tumor location, endoscopic obstruction, and CEA levels with CT indicators including CT value, short-axis and long-axis diameter of lymph nodes, achieving an AUC of 0.80 (48). This predictive model can help the surgical decision of whether to perform high or low ligation of the inferior mesenteric artery (IMA).

An alternative approach shifted from node-level morphology to distribution patterns. Guan et al. proposed a staging method based on lymph node distribution for MSI-H colon cancer in a retrospective multi-center study, defining vascular distribution as negative and jammed cluster/partial fusion as positive. It distinguished metastatic from reactive hyperplastic lymph nodes, improving diagnostic accuracy from 46% to 90% with a specificity of 97% (49).

Another pathway employed functional imaging with radiomics. Hao et al. found that a b-value of 2,000 provided the optimal DWI image quality, and the radiomics model based on this sequence was able to identify microstructural features of the tumor for patient-level prediction of LNM in a retrospective single-center study, achieving an AUC of 0.728 (50).

Accurate LNM diagnosis guides neoadjuvant therapy, lymphadenectomy extent, vascular ligation, and adjuvant chemotherapy. Yet from a surgical perspective, the imaging-pathology co-registration challenge may be of secondary practical relevance, as surgeons perform regional lymphadenectomy along vascular pathways, not node-by-node excision. What matters is the overall risk distribution within lymphatic drainage territories, not the exact correspondence of individual nodes. Refining imaging-pathology correlation remains an important research direction for elucidating metastatic mechanisms, but from a surgical standpoint, the more actionable question is the overall risk distribution within lymphatic drainage territories rather than the exact correspondence of individual nodes.

### 3.3 Distant metastasis

For distant metastasis diagnosis, AI has shown suboptimal sensitivity. In a retrospective single-center study, a DL model developed by Kim K et al. for detecting colorectal liver metastases (CRLM) on CT achieved a lesion-level sensitivity of only 56.5% for subcentimeter metastases, with a false-positive rate 3.7 times higher than radiologists (51). Such performance limits clinical trust, as both missed small metastases and overcalling benign lesions have significant consequences. Such a high false-positive rate may lead to unnecessary examinations or overtreatment, highlighting that current DL algorithms still struggle to balance sensitivity and specificity. Li et al. further developed a DL model using portal-venous phase CT to differentiate CRLM from hepatic hemangiomas in a retrospective multi-center study. Their ResNet-152 model achieved an AUC of 0.776 in the test set for distinguishing between the two entities. Subgroup analysis revealed that DL assistance significantly improved radiologists’ diagnostic performance for 10–30 mm lesions, with the AUC increasing from 0.851 to 0.879, but provided no significant benefit for subcentimeter lesions (≤10 mm) (52). Compared with endoscopy and surgery, radiological diagnosis is predominantly performed on static images with lower dynamism, facilitating stable AI feature extraction and improved efficiency.

Despite feasibility, clinical translation is hindered. Poor model interpretability and evaluation metrics rarely linked to clinical outcomes make it difficult to quantify true clinical value.

## 4 Surgical intervention: the application on intraoperative procedure

AI-assisted intraoperative navigation has demonstrated technical feasibility in colorectal surgery, offering the potential to enhance surgical precision and support intraoperative decision-making. By providing real-time analysis of surgical videos, AI systems can support surgeons in recognizing critical anatomical structures and optimizing dissection planes, it can also function to accelerate the learning curve of junior surgeons. Current researches mainly focus on surgical workflow, instruction recognition and anatomical structure segmentation. An overview of representative studies is summarized in **Table 2** (53–75).

**Table 2:** Current artificial intelligence applications on colorectal cancer intraoperative procedure.

Category	Author	Study design	Sample size	Validation type	Surgical procedure	Target structure	Model	Performance
Surgical Workflow	(53)	Retrospective/Multi-center	300 videos, 82M frames	Internal (holdout)	Laparoscopic sigmoidectomy	7 surgical phases, actions (extracorporeal action, irrigation)	Inception-ResNet-v2 + LightGBM	Accuracy: 91.9% (phase), 89.4% (extracorporeal action), 82.5% (irrigation action);
	(54)	Retrospective/Single-center	71 cases, 8.7M frames	Internal (holdout)	Laparoscopic sigmoid colectomy/high anterior resection	9 surgical phases, 3 actions (dissection/exposure/other), 5 tools	Xception (phase/action) + U-Net (tool segmentation)	Accuracy: 81.0% (phase), 83.2% (action classification), mIoU for tools: 51.2%
	(55)	Retrospective/Single-center	50 videos	Internal (holdout)	Transanal total mesorectal excision	5 main surgical phases (with 2 steps further divided into 4 sub-steps each)	Xception	Accuracy: 93.2% (main steps), 78% (sub-step)
	(56)	Prospective/Single-center	57 videos	Internal (crossvalidation)	Robot-assisted rectal resection	5 surgical phases and 16 target structures	Phase: ResNet50 + LSTM/ MSTCN/Trans-SVNet; Segmentation: DeepLabv3 with ResNet50	Phase: F1: 0.82 ± 0.01, Accuracy: 0.84 ± 0.03; Segmentation: mIoU: 0.14 ± 0.22 to 0.80 ± 0.14 (organ/tissue), 0.11 ± 0.11 to 0.44 ± 0.30 (dissection area)
	(57)	Retrospective/Single-center	78 videos, 13.1M frames	Internal (holdout)	Laparoscopic right-sided colectomy	8 surgical steps combined into 5 phases	Xception (pretrained on ImageNet)	Accuracy: 72.1% (8-step), 82.9% (5-step)
Nerves & Vessels	(58)	Retrospective/Single-center	245 HGN videos, 44 SHP videos	Internal (crossvalidation)	Laparoscopic left-sided colorectal resection	HGN/SHP	DeepLabV3+ with Xception	Dice: 0.56± 0.03 (HGN), 0.49± 0.07 (SHP); Model recognized HGN earlier than surgeon in 50% of cases
	(59)	Retrospective/Single-center	1,424 training, 356 validation images	Internal (holdout)	Laparoscopic total mesorectal excision	Pelvic autonomic nerve	DeepLabv3+ with ResNet50_Vd	F1: 0.7011; mIoU: 0.75 Precision: 0.7494; Recall: 0.6587;
	(60)	Prospective/Single-center	51 surgeries, 101 scenarios	External (realtime intraoperative validation)	Laparoscopic left-sided colorectal resection	HGN, lumbar splanchnic nerves, pelvic visceral nerve	Eureka (AI navigation system)	Recognition assistance rate: 43.2% (right HGN), 48.1% (left HGN), 90.6% (right lumbar splanchnic nerves), 88.6% (left lumbar splanchnic nerves), 20.7% (pelvic visceral nerves)
	(61)	Retrospective/Multi-center	60 videos, 1,200 images	Internal (crossvalidation)	Laparoscopic sigmoid colon resection/high anterior resection	Inferior mesenteric artery	DeepLabv3+ with ResNeSt-269	Mean dice similarity coefficient: 0.798; Max dice similarity coefficient: 0.816
	(62)	Retrospective/Multi-center	43 videos, 1,625 images	Internal (crossvalidation)	Laparoscopic right hemicolectomy	GCT, superior mesenteric vein	DeepLabV3+ with Xception	Dice: 0.84 (vein), 0.61 (GCT, superior mesenteric vein, and background distinguished), 0.58 (GCT distinguished from the superior mesenteric vein and background)
	(63)	Retrospective/Single-center	22 videos, 992 images	Internal (crossvalidation)	Laparoscopic lateral lymph node dissection for rectal cancer	Obturator nerve, external iliac vein, external iliac artery	Deep learning-based semantic segmentation	Dice: 0.789 (obturator nerve), 0.736 (external iliac vein), 0.574 (external iliac artery)
	(64)	Retrospective/Multi-center	70 videos, 740 images	Internal (holdout)	Laparoscopic/Robot-assisted rectal cancer surgery	Nerve-like structures (hypogastric & pelvic splanchnic nerves)	U-Net (segmentation)	Dice: 0.442; IoU: 0.292; Usefulness score (expert): 3.38/4
Ureter	(65)	Retrospective/Single-center	252 ureter videos, 194 nerve videos	Internal (holdout)	Laparoscopic left-sided colorectal resection	Ureter, hypogastric nerves, aortic plexus	UreterNet & NerveNet (Feature Pyramid Networks + EfficientNetB7/OCRNet + HRNet)	Dice: 0.722(ureter), 0.579 (hypogastric nerve), 0.628 (aortic plexus); AI faster than surgeon in 75% of cases (ESSQS-unqualified: 84%, qualified: 66%)
	(66)	Retrospective/Single-center	86 videos, 1,237 images	Internal (crossvalidation)	Laparoscopic sigmoidectomy	(Real-time) Left ureter	YOLO-based deep learning model	Precision: 0.94; Recall: 0.88; Dice: 0.90; 32FPS
Excision plane	(67)	Retrospective/Single-center	32 videos, 600 images	Internal (holdout)	Laparoscopic left-sided colorectal resection	Areolar tissue	DeepLabv3+	Dice: 0.84
	(68)	Retrospective/Single-center	157 videos, 2,861 images	Internal (crossvalidation)	Laparoscopic TME	Mesorectal fascia (danger zone – blue), parietal pelvic fascia (danger zone – red), holy plane (safe zone – green)	Feature Pyramid Networks + EfficientNetB7	Dice: 90.4% (blue), 90.6% (red),68.5% (green) NSD: 70.4%, 73.2%, 54.6%
Anastomotic perfusion assessment	(69)	Retrospective/Single-center	Not specified	Internal (holdout)	Laparoscopic left-sided colorectal resection	microcirculation risk (safe/intermediate/dangerous)	SOM	F1 score: 0.75 (AI) vs 0.44–0.67 (conventional parameters); Processing time: 48.03s; AUC: 0.842
	(70)	Prospective/Single-center	20 training, 28 test videos	Internal + Realtime intheater	Laparoscopic/robotic colorectal resection	ICGFA timing features; stapler placement zone (expert/good/poor)	bi-LSTM (sequence classification) + ML (KNN)	DL object-level accuracy: 100% (in-theater); Dice: 0.81–0.85; Recall: 0.98; Prediction time: around 13s
	(71)	Retrospective/Multi-center	26 patients, 5,356 frames	Internal (holdout)	Laparoscopic/robotic colorectal resection	Anastomosis video frames	CNN (EfficientNetB0, B7, ResNet50, MobileNetV2)	Accuracy: 99.5%; AUC: 0.996; Sensitivity: 99.2%; Specificity: 100%;
Surgical skill assessment	(72)	Retrospective/Multi-center	74 videos, 1,480 clips	Internal (Leave-One-Site-Out)	Laparoscopic sigmoidectomy/high anterior resection	Surgical skill level (ESSQS score groups: low/middle/high)	3D CNN (Inception-v1 I3D)	Accuracy: 75.0% (3-class); Sensitivity (low-skill screen): 94.1%; Specificity (low-skill): 96.5%; AUC (low-skill): 0.989
	(73)	Retrospective/Multi-center	60 training, 60 validation videos	Internal (holdout)	Laparoscopic sigmoidectomy	Standardized surgical field development	Xception + Softmax	Spearman’s r (AICS vs ESSQS): 0.81;AUC (low-skill screen): 0.93; AUC (high-skill screen): 0.94
Surgical efficacy assessment	(74)	Retrospective/Multi-center	30 training, 57 test videos	Internal (holdout)	Laparoscopic sigmoidectomy	Dissection (D)/Exposure (E)/Invalid time; D/E ratio; D-E transition	Xception (image classification)	F1: D-0.92, E-0.82, I-0.74; D-E transition (low-skill): 405 vs high-skill: 204 (p<0.01)
	(75)	Retrospective/Multi-center	766 videos, 1.15M test frames	Internal (holdout)	Laparoscopic sigmoidectomy/high anterior resection	Efficient-dissection time ratio (monopolar device)	EfficientNetB7 (white-light imaging + optical flow ensemble)	Overall accuracy: 0.91; F1 (dissection-on): 0.58; correlation (auto vs manual): r=0.542 (p<0.001)

LightGBM, Light Gradient Boosting Machine; mIoU, mean Intersection over Union; LSTM, Long Short-Term Memory; MSTCN, Multi-Stage Temporal Convolutional Network; HGN, Hypogastric Nerve; SHP, Superior Hypogastric Plexus; GCT, Gastrocolic Trunk; IoU, Intersection over Union; OCTNet, Object-Contextual Representation Network; HRNet, High-Resolution Network; NSD, Normalized Surface Distance; SOM, Self-Organizing Map; IGCFA, ndocyanine Green Fluorescence Angiography; bi-LSTM, Bidirectional Long Short-Term Memory; KNN, K-Nearest Neighbors; CNN, Convolutional Neural Network; ESSQS, Endoscopic Surgical Skill Qualification System; TME, Total Mesorectal Excision; AUC, Area Under the Curve; AICS, AI Confidence Score.

The AI models for surgical navigation summarized above are based on DL and remain at the research prototype stage, without regulatory approval or prospective deployment. These studies reveal a clear performance hierarchy in AI-based anatomical segmentation. For larger, well-defined landmarks such as the inferior mesenteric artery and mesorectal fascia, several studies have reported promising Dice coefficients, with some exceeding 0.90, suggesting that AI can reliably identify these gross anatomical targets. Studies focusing on finer, functionally critical structures such as autonomic nerves and the ureter have generally reported lower Dice coefficients, often in the range of 0.44 to 0.72, highlighting the greater technical challenges posed by these targets. Notably, the reported performance for nerve recognition varies considerably even for the same or similar structures, from 0.442 to 0.789 depending on the specific nerve type, surgical field, and study population, suggesting that AI’s ability to recognize these structures is highly context-dependent. This inconsistency reflects fundamental challenges that fine structures are inherently more difficult to annotate consistently, exhibit greater anatomical variability across patients, and are more susceptible to intraoperative factors such as bleeding, smoke, and tissue manipulation. Furthermore, the clinical utility of these models cannot be inferred from Dice coefficients alone. Kinoshita et al. found that even with a Dice of only 0.442, expert surgeons rated the model’s clinical usefulness highly, underscoring the disconnect between pixel-level segmentation metrics and intraoperative decision support (64). Crucially, nearly all available evidence leaves the generalizability of these results to broader clinical settings, different surgical platforms, and more challenging anatomical conditions unproven. Likewise, prospective evidence demonstrating definitive improvements in complications or functional outcomes remains limited and requires further development. Apart from accuracy, real-time capability is another crucial consideration for practical application. Current studies demonstrate that many AI models now achieve near-real-time processing speeds, with inference times as low as 0.08 seconds per frame. For instance, Kojima et al. demonstrated real-time segmentation of autonomic nerves during laparoscopic colorectal surgery at 12.5 fps (58), while Sasaki et al. achieved 12.7 fps for vessel and nerve recognition during lateral lymph node dissection (63), and Han et al. further showed real-time pelvic autonomic nerve and surgical plane recognition during TME (59). These technical advances suggest that computational speed is becoming less limiting. However, they still operate under relatively favorable conditions where target structures are already adequately exposed and visible. What surgeons truly need is predictive navigational guidance that integrates preoperative imaging data, such as CT and MRI, to project the anticipated locations of critical anatomical structures onto the intraoperative field in real time, thereby preventing inadvertent injuries before structures are even visualized (**Figure 1**). This paradigm would shift AI from a passive viewer of visible anatomy to an active anticipatory guide, yet such integrated approaches remain largely unexplored.

**Figure 1:**
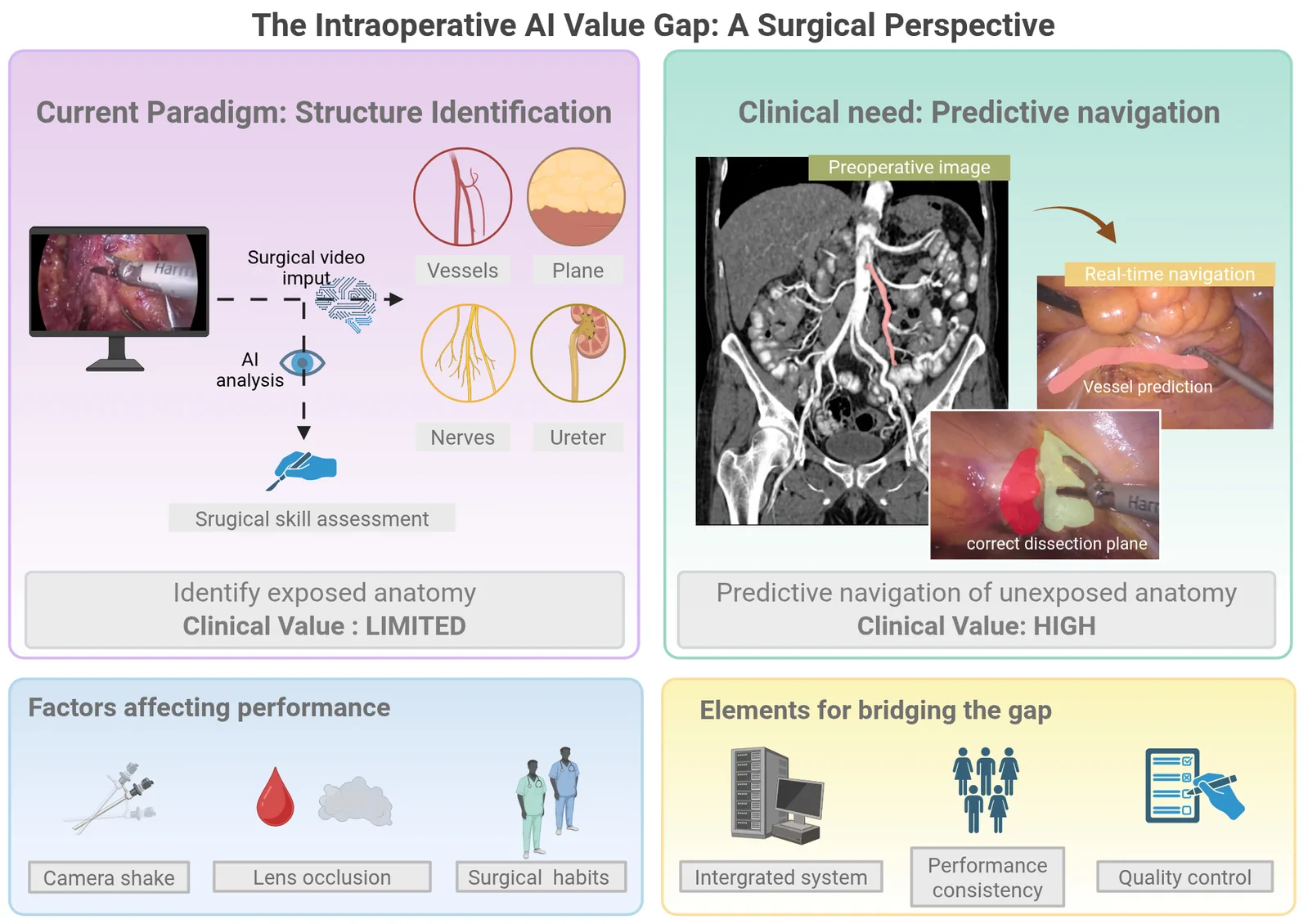
Surgical perspective on the AI application value gap in intraoperative procedures. All clinical images were obtained from the authors’ institutional database and are used with permission.

Another medical decision-support technology, AI-assisted anastomotic perfusion assessment, offers value to surgeons as well. Currently, anastomotic assessment relies primarily on subjective surgeon judgment, including visual inspection of bowel color, edema, and perfusion at the anastomotic margins. Park et al. developed an AI-based real-time microcirculation analysis system using a self-organizing map network to classify ICG fluorescence intensity curves into 25 patterns for intraoperative color-coded risk mapping in a retrospective study, performing the feasibility of anastomotic complications prediction (69). Singaravelu et al. advanced this field with a DL model in a prospective study that analyzes ICG signal dynamics, including intensity rise rate, peak intensity, and washout patterns, to replicate expert perfusion interpretation in real time with pathological confirmation (70). Taking a different approach, Mascarenhas et al. developed a convolutional neural network that analyzes intraoperative video frames of the anastomosis itself, rather than perfusion signals, to predict leakage directly from visual tissue features and generate heatmaps highlighting high-risk areas (71). However, variability in camera distance, lighting, tissue motion, and ICG injection dynamics complicates intraoperative validation. Black-box models further undermine surgeon trust, as understanding the rationale behind AI recommendations is critical for irreversible surgical decisions. Moreover, beyond perfusion, tension, technical precision, and tissue quality also contribute to anastomotic leakage, suggesting that future models should integrate a broader range of intraoperative data.

Despite the promising potential of AI application in surgery, translation from technical feasibility to routine practice faces multiple barriers. Most models remain research prototypes without regulatory approval. Integration is hindered by a lack of standardized formats and device interoperability. Real-time processing demands high-performance hardware that many institutions lack. Most models target isolated tasks, whereas actual surgery requires simultaneous identification of multiple structures. Prospective evidence of improved outcomes is lacking, and proof of complication reduction or functional benefit is required before clinical application. Surgeon acceptance is further limited by liability concerns, workflow disruption, and cognitive load from additional on-screen information.

While the above studies focus on intraoperative decision support, AI also holds promise for operation quality assurance through automated video-based skill assessment. Currently, surgical quality evaluation relies on manual review using tools like OSATS (Objective Structured Assessment of Technical Skills) and GOALS (Global Operative Assessment of Laparoscopic Skills), which depend on subjective expert judgment with only moderate interobserver agreement and are resource-intensive. The lack of standardized consensus further leads to inter-institutional heterogeneity, limiting comparability and scalability. AI offers a potential solution. Kitaguchi et al. developed a model that analyzes spatiotemporal video features to classify surgical clips into three proficiency levels with approximately 75% accuracy, assessing overall surgical flow and instrument handling (72). Igaki et al. took a different approach, training a model to recognize standardized surgical field development and outputting an AI confidence score that strongly correlated with established skill metrics (AUC 0.93–0.94) (73). Both demonstrated the feasibility that AI can capture technical proficiency from video data alone, without sensors or manual annotation.

However, surgical quality assessment remains underexplored compared to diagnostic AI. Robust quality assessment could make expert standards explicit and measurable, provide objective feedback, and enable competency-based credentialing. Yet current efforts fall short. Most studies reduce surgical quality to a binary high-versus-low skill classification rather than a multidimensional construct encompassing decision-making, tissue handling, efficiency, and adaptability. The resulting metrics, such as confidence scores, are surrogate markers with unclear clinical meaning. A low score offers no actionable guidance. Moreover, training on expert-defined labels may entrench existing biases rather than reveal new insights.

## 5 Postoperative management: complication prediction, prognosis prediction and follow-up treatment guidance

### 5.1 Complication prediction

Postoperative complications, especially anastomotic leakage (AL), is one of the most severe adverse events after CRC surgery. They are associated with prolonged hospital stays, higher reoperation rates, delayed adjuvant therapy, and worse long-term oncological outcomes. Traditional risk prediction models struggle to capture complex nonlinear relationships, whereas AI-assisted models can incorporate nonlinear interactions and have been shown to improve predictive accuracy. A meta-analysis by Mohamedahmed et al. further confirmed that machine learning (ML) performs well in predicting AL, mortality, surgical site infection, and prolonged hospital stay (76).

Current studies can be classified into preoperative prediction, intraoperative real-time prediction and early postoperative prediction.

Preoperative prediction models have been developed using various ML/DL approaches. Reported performance varies considerably depending on the outcome and study population, with AUCs ranging from 0.618 for surgical site infection to 0.88 for AL in selected studies (77–84) (**Table 3**). Notably, these studies span diverse prediction targets, from anastomotic leakage to mortality, demonstrating the broad applicability of ML across postoperative outcomes. But most current studies rely solely on AUC and accuracy, with calibration, decision curve analysis, and clinical utility assessment rarely reported. This reflects a general gap in the field rather than a limitation of any individual study. Several critical observations emerge from a technical and clinical perspective as well. Technically, most models are trained on routine data but omit surgery-specific variables such as anastomotic technique, tissue quality, and intraoperative events, which surgeons intuitively recognize as critical determinants of AL. Predictor selection varies widely across studies, with no consensus on which variables matter most, and inter-institutional differences in variable definitions further complicate cross-center deployment and generalizability. Most models rely on internal validation, and when external validation is performed, performance often drops markedly, as demonstrated by Taha-Mehlitz et al. (AUC from 0.78 internally to 0.60 externally) (82). Nonetheless, some models have shown more robust external performance. Ochs et al.’s cross-attention meta-model achieved a more robust external F1 of 70% across 13 centers (84) and Lu et al. developed a rare standalone clinical application (83), representing advances in the field. By contrast, preoperative prediction in other gastrointestinal malignancies has progressed further in methodological rigor and clinical integration. The DeepComp model, developed for gastric cancer, integrates preoperative CT imaging and clinical variables to predict major complications and overall survival. Validated across 5,237 patients from 11 centers (AUC 0.824–0.869 externally), it outperformed nine clinical risk scores and improved surgeon sensitivity from 47.1% to 87.9% when used as an assistive tool. A prospective multi-center validation study is ongoing. (85).These features, including prospective validation, workflow integration, multimodal data fusion, and quantifiable human-AI synergy, are absent in most current CRC models.

**Table 3:** Current artificial intelligence applications on colorectal cancer postoperative complication prediction based on preoperative prediction models.

Author	Type of study	Sample size (Cases)	Validation type	Model	Prediction target	Key predictors	AUC
(77)	Retrospective/Single-center	13,399	Internal (10-fold CV)	GBM vs. LR	Postoperative bleeding (within 7 days)	Anemia, hemophilia, nutrition, weakness, mobility, activity level, operative time, heart failure, kidney disease	0.822(GBM) 0.735(LR)
(80)	Retrospective/Multi-center	23,907	Internal	LASSO LR	Severe complications (≥IIIb), AL	ASA ≥3, emergency surgery, open surgery, tumor perforation, total colectomy, female sex, right-sided colectomy, BMI	0.704 (complications) 0.690 (AL)
(79)	Retrospective/Multi-center	65,612	Internal	LASSO LR	30/90/180-day mortality	Age, ASA, emergency surgery, WHO performance status, CCI, albumin, BMI, tumor stage, surgical procedure	0.871 (30d) 0.874 (90d) 0.876 (180d)
(81)	Retrospective/Single-center	328	Internal	C3D DL	Multiple stapler firings (≥3)	CEA >5 ng/mL, tumor size ≥5 cm, pelvic MRI features (pelvis shape, mesorectum thickness, tumor infiltration), BMI, sex	0.88 (integrated) 0.81 (image-only) 0.72 (clinical)
(78)	Retrospective/Multi-center	275,152	Internal (temporal split)	NN vs. XGB vs. RF vs. LR	Surgical site infection (SSI)	Organ-space SSI PATOS, operative time, oral antibiotic bowel prep, surgical approach, BMI, ASA, age	0.769(NN) 0.766(XGB) 0.764(RF) 0.677(LR)
(82)	Retrospective/Multi-center	1,442	Internal (5-fold CV) + External	RF vs. LR	AL	Surgical procedure, emergency surgery, renal function, preoperative leukocytosis, steroid use, smoking, liver metastasis, alcohol, ASA	0.78/0.69 (internal); 0.60/0.61 (external)
(84)	Retrospective/Multi-center	9,120	Internal + External	Cross-attention meta-model (CatBoost, LightGBM),	AL	Logits of 4 base models, CRP, CCI, prior abdominal surgery, albumin, BMI, indication, surgical procedure, surgeon experience, emergency surgery	CV F1: 87% External F1: 70%
(83)	Prospective/Multi-center	3,926	Internal + External	RF	Overall complications, infectious complications	BMI, examined lymph nodes, age, comorbidity of infection, sex, ASA, emergency surgery, surgical procedure, neoadjuvant therapy, pT/pN stage, combined resection	0.644 (overall) 0.631 (severe) 0.618 (surgical infection)

AUC, Area Under the Curve; GBM, Gradient Boosting Machine; LR, Logistic Regression; NN, Neural Network; XGB, Extreme Gradient Boosting; RF, Random Forest; C3D, Convolutional 3D; DL, Deep learning; AL, Anastomotic Leakage; ASA, American Society of Anesthesiologists; BMI, Body Mass Index; WHO, World Health Organization; CCI, Charlson Comorbidity Index; CRP, C-reactive Protein; CEA, Carcinoembryonic Antigen.

For intraoperation, real-time prediction provides direct decision support. Three models have been introduced previously (69–71). They offer a more clinically relevant approach by assessing anastomotic perfusion and tissue quality at the critical moment of decision-making, complementing subjective surgeon assessment with objective, data-driven perfusion analysis. However, the clinical trade-off between false positives (unnecessary stomas) and false negatives (missed leaks) remains unresolved, limiting adoption.

Early postoperative prediction leverages dynamic changes in laboratory markers. Adams and Papagrigoriadis used preoperative elements and postoperative CRP, platelet count, and hemoglobin to predict AL with an AUC of 0.89 in a retrospective single-center study (86), while Agnes et al. achieved an AUC of 0.779 using day 1–2 markers in a retrospective single-center study, improving to 0.811 with day 3 data (87). Early postoperative prediction still facilitates timely intervention such as drainage or stoma creation, but its overall clinical value is less substantial than preoperative or intraoperative prediction, given that it does not guide the primary surgical decision. What surgeons truly need is prediction that occurs before or during surgery, identifying high-risk patients preoperatively to optimize surgical planning, or assessing anastomotic integrity intraoperatively to guide stoma construction or anastomotic reinforcement. Therefore, its translational value falls short of what the reported AUC might suggest and its clinical benefit remains to be validated.

### 5.2 Risk stratification assisting adjuvant chemotherapy

Beyond short-term complication prediction, AI has been applied in long-term risk stratification and prognosis prediction. Current research has evolved from single-modality, traditional machine learning approaches toward multimodal integration and biomarker-driven frameworks.

Risk stratification to inform adjuvant chemotherapy (ACT) decisions represents a clinically actionable application of AI in postoperative CRC management, particularly for CRC stage II disease where the benefit of ACT remains controversial and overtreatment carries substantial morbidity.

Histopathology-based DL models have since refined this approach by stratifying patients directly from digitized tumor slides. The HIBRID study by Loeffler et al. demonstrated that a DL histopathology model on a retrospective cohort and validated on the prospective cohort, demonstrating that it could further stratify Minimal residual disease (MRD) -negative patients, with only high-risk patients derived significant benefit from ACT (HR 0.49, p = 0.01) (88). Similarly, the DL-RRS model developed by Xiao et al. in a retrospective multi-center study classified patients based on histomorphological features extracted from whole-slide images, achieving a 5-year RFS separation of 45.7% versus 82.5% between high- and low-risk groups, and confirming that ACT benefit was confined to the high-risk stratum (HR 0.15, p < 0.001) among stage II patients but not in the low-risk group. (89). The SurvFinder developed by Zhao et al. identified tertiary lymphoid structures (TLSs) as key prognostic features in stage II CRC using a multi-view DL approach in a retrospective multi-center study, stratifying patients across external cohorts with AUROCs of 0.805–0.827 (90). Multimodal systems have further improved stratification granularity by integrating complementary data sources. The IRIS-CRC system by Huang et al. integrated CT-based DL with pathological markers to create a four-tier risk stratification for stage II CRC in a retrospective multi-center study, reclassifying 27.1% of guideline-defined high-risk patients to favorable prognosis and 6.5% of low-risk patients to very poor prognosis (91). The MTMSCRC signature by Jiang et al. in a retrospective multi-center cohort, integrated pathomics, collagen features, and CD3/CD8 immune scores, stratifying patients into three risk groups with 5-year DFS rates of 92.0%, 59.8%, and 2.9%, respectively; only high and medium-risk patients derived chemotherapy benefit (92). At the transcriptomic level, the PIANOS classifier by Cai et al., developed in a retrospective multi-center, used gene expression data from 24 cohorts to stratify patients by pathway-level activation patterns, achieving an overall C-index of 0.74. Low-risk patients showed greater sensitivity to standard chemotherapies, whereas high-risk patients with enhanced angiogenesis pathway activation derived significant OS benefit from bevacizumab-containing regimens, suggesting that AI-driven transcriptomic stratification has the potential to inform both chemotherapy and targeted therapy selection, though this finding requires further prospective validation (93).

In addition to the histopathology-based models mentioned above, several pathology foundation models have demonstrated potential in CRC risk stratification and adjuvant therapy guidance. CTransPath is a hybrid CNN-Swin Transformer pretrained on large-scale histopathology images via semantically-relevant contrastive learning, serving as a general-purpose feature extractor for pathological tasks (94). It has been employed as a feature extractor in a large-scale retrospective multi-center study (>13,000 patients, 16 cohorts), where a Transformer-based aggregation model achieved high accuracy for MSI (AUROC 0.97), BRAF (AUROC 0.88), and KRAS (AUROC 0.80) prediction from routine H&E-stained slides. The model trained on surgical resection specimens generalized successfully to endoscopic biopsies (AUROC 0.92), demonstrating stable predictive performance and the feasibility of rapid molecular profiling from biopsy material to inform neoadjuvant immunotherapy decisions (95). Likewise, a general-purpose weakly supervised model named CHIEF, which integrates CTransPath (94) as its image encoder within a weakly supervised learning system, was pretrained on over 60,000 whole-slide images (WSI) across 19 anatomical sites and has been validated in colorectal and other gastrointestinal cancers. It showed robust performance in predicting microsatellite instability status (MSI) directly from routine H&E-stained slides in CRC, with AUROCs of 0.869–0.875 across multiple cohorts (96). PROGPATH is a pancancer prognosis model integrating histopathological images with clinical variables (age, sex, stage), which validated across 17 independent cohorts covering 12 cancer types (>7,300 WSIs, >4,400 patients). In CRC, it achieved consistent survival stratification and identified patients likely to benefit from ACT, with significant survival separation in pharmacotherapy and radiotherapy subgroups. Although based on histopathological images and lacking prospective validation, these models represent a complementary approach to refine ACT decisions without molecular assays (97). These foundation models represent a notable advance in computational pathology, offering clinically actionable insights from routine slides without extra molecular testing.

Beyond these specific models, several broader considerations merit attention when contemplating the clinical integration of AI-based risk stratification tools. The clinical challenge is often deciding to withhold chemotherapy, not to give it. Guidelines provide clear indications for high-risk patients, but low-risk patients lack objective evidence to justify forgoing treatment. AI-based low-risk prediction fills this gap by offering quantitative, interpretable evidence for de-escalation, reflecting the essence of precision medicine. In practice, surgeons prefer simple, explainable outputs over complex black-box models. An interpretable model with moderate AUC may offer greater clinical value than a superior but opaque one. However, most studies supporting these models are retrospective, and even when performance appears promising, such findings cannot be equated with clinical efficacy without prospective validation. Direct evidence linking AI-based stratification to improved patient outcomes remains limited. Despite these limitations, the clinical potential of these tools is clear, and their value ultimately depends on whether they can help clinicians make better decisions, not just generate better statistics. Practical considerations, such as timing, also affect clinical application. Postoperative models support adjuvant decisions, while preoperative tools inform surgical planning. Patient acceptance is equally important. Although AI provides objective evidence for treatment de-escalation, patients trust physicians more than algorithms. Clinician endorsement and clear communication of AI results are therefore essential for patient adherence and shared decision-making.

### 5.3 Risk stratification for prognosis prediction

Beyond chemotherapy decisions, AI-based risk stratification has been explored to provide more precise survival prediction and support the selection of other therapeutic modalities. Critically, the ultimate test of any prognostic model is whether it can translate predicted risk into actionable interventions that improve patient outcomes.

For multimodal level, the PRISM-CRC framework by Shi et al. integrated histopathology, CT imaging, and clinical data to predict 5-year DFS with a C-index of 0.82 in a retrospective multi-center study (98). The HydraMamba framework by Wang et al. unified endoscopy and CT through a Mamba state-space architecture, achieving a C-index of 0.832 for survival prediction with favorable calibration in a retrospective multi-center study (99). The HMTsurv framework by He et al. integrated histopathology, transcriptomics, and microbiome data, identifying 14 pan-cancer survival biomarkers including MAGE family genes consistently upregulated in high-risk subgroups in a retrospective study using multi-cohort data (100). The DL-RRS model developed by Xiao et al. achieved AUCs of 0.833 internally and 0.715 externally for predicting 5-year recurrence, achieving higher performance than conventional clinicopathological factors in a retrospective multi-center study. Notably, integrating DL-RRS with clinical data did not significantly improve performance, suggesting that histology-based deep learning already captures much of the prognostic information contained in clinical variables (89). The SurvFinder framework developed by Zhao et al. further demonstrated that multi-view integration of TLS characteristics consistently predicted prognosis across multi-center datasets in a retrospective multi-center study, with MVNet achieving AUROCs of 0.805–0.827, showing higher performance than traditional clinicopathological prognostic parameters (HR 8.23, p < 0.001) (90).

In addition to these CRC-specific models, several pathology foundation models have been developed or adapted for prognosis prediction across gastrointestinal and other cancer types. PROGPATH, as introduced before, has also demonstrated prognostic capability, achieving a mean C-index of 0.731 in the TCGA held-out sets and 0.727 in the PLCO cohorts, while maintaining significant survival stratification across treatment subgroups and biomarker-defined subgroups, highlighting its robustness and potential for translation to real-world clinical settings (97). CHIEF similarly demonstrated survival stratification across multiple cancer types including CRC, with a mean C-index of approximately 0.74, successfully distinguishing longer-term from shorter-term survivors in both early- and advanced-stage patients (96).

While these studies demonstrate prognostic power, they are mostly retrospective in nature and their findings have not yet been prospectively validated. In contrast, a prospective study by Rosen et al. provides a compelling example of translation to clinical practice. Using Danish nationwide registry data from 18,403 patients, they developed a LASSO logistic regression model to predict 1-year mortality (external validation AUC 0.79) and stratified patients into four risk tiers with gradually strengthening perioperative interventions (101). In a before/after cohort study, severe complications decreased from 28.0% to 19.1% (adjusted OR 0.63, p = 0.02), and medical complications dropped from 37.3% to 23.7% (OR 0.53, p < 0.001). Cost-effectiveness analysis estimated savings of approximately US $2,848 per patient. This study represents one of the few fully deployed AI-based decision support tools in colorectal surgery, demonstrating that risk stratification can improve outcomes when linked to standardized intervention bundles.

The RSA model in gastric cancer also illustrates how some of these conditions can be addressed, particularly regarding prospective design and interpretability. Developed from 1,763 patients and validated across multi-center and prospective cohorts (NCT01516944), it integrates histopathological and clinical features for recurrence stratification (AUC 0.887) (102). Beyond performance metrics, it demonstrates survival separation across risk groups and provides SHAP-based interpretability, which offered a useful template for CRC risk stratification.

Apart from model performance and validation, a more fundamental question is what constitutes clinically useful AI in the first place. Most models produce statistical risk scores, but a score alone does not constitute a treatment decision. Only a few studies have linked risk stratification to standardized interventions and demonstrated measurable outcome improvements. For AI to deliver clinical value, some conditions must be met: Predictions must be available within the clinical decision window; otherwise, even the most accurate model offers no practical utility. Outputs must integrate seamlessly into existing workflows rather than requiring surgeons to adapt to the technology. Models must also provide interpretability, enabling clinicians to understand the basis of predictions, build trust, and make informed decisions. AI-driven decisions should demonstrate improved patient outcomes over conventional approaches, not merely statistical model optimization.

## 6 Discussion

### 6.1 Challenges of AI in practical clinical application

Despite AI’s promise across CRC management, a substantial gap remains between technical feasibility and clinical adoption. Understanding this gap requires examining the whole procedure of AI system development and deployment across technical validation, clinical validation, regulatory authorization, and post-deployment monitoring.

Technical validation is the first stage, in which AI models are typically developed and tested on retrospective, often single-center datasets. Most current CRC AI models remain at this stage, trained on limited datasets and facing multiple challenges, including data heterogeneity and scarcity, class imbalance, insufficient annotation quality, and privacy restrictions on data sharing. These factors collectively constrain model generalizability. When models proceed to external validation, differences in scanner or endoscope manufacturers, imaging protocols, institutional practices, demographic composition, and disease prevalence may further induce dataset shift, leading to substantial performance degradation. Importantly, variations across healthcare systems in patient composition, clinical pathways, and disease spectrum also represent critical sources of dataset shift. In particular, differences in healthcare quality and capacity such as diagnostic accuracy, treatment standardization, physician expertise, and equipment maintenance practices, can introduce additional variability. For example, AI models developed using high-quality, standardized imaging data from well-resourced centers may underperform when applied to institutions with less consistent imaging protocols, outdated equipment, or greater reader variability. These factors profoundly affect model applicability across different clinical scenarios. However, subgroupspecific performance across different patient demographics or disease subtypes has rarely been systematically evaluated in the current literature, making it difficult to determine whether models trained in selected institutions can be safely deployed in broader clinical settings. Meanwhile, the use of inconsistent performance metrics across studies and the lack of standardized evaluation frameworks and most published models also lack calibration or decision curve analysis, further complicate meaningful comparisons and limit the assessment of their real-world clinical utility. More fundamentally, high technical performance such as AUC or Dice coefficients does not automatically equate to improved patient outcomes, as most studies rely on surrogate endpoints rather than clinically meaningful measures such as complication reduction or survival benefit; this gap underscores the urgent need for prospective, interventional studies that directly validate whether AI-assisted management translates into tangible patient benefit.

While these challenges are widely recognized across medical AI, several cross-disciplinary methodological advances may offer potential solutions. FMDet uses Fourierbased augmentation and semantic segmentation to improve crossdomain generalizability (103), which could help CRC models maintain performance across different imaging protocols and scanner vendors. HiCervix provides a hierarchical classification framework for finegrained typing and error severity control (104), offering a strategy for CRC staging where misclassification severity varies by stage. Aegis introduces feature alignment to mitigate domain shift (105), addressing the variability in CRC imaging data across institutions. HPDT delivers interpretable stepwise predictions that prioritize less severe errors (106), aligning with the graded clinical decisions in CRC management. UADFM combines causal testtime adaptation with uncertainty decomposition to enhance robustness and confidence calibration (107), which could help clinicians identify cases requiring closer review in CRC staging. While developed outside CRC, these approaches, including domain generalization, hierarchical interpretability, and uncertainty quantification, could help address annotation bottlenecks and crosscenter heterogeneity limiting CRC model translation.

Clinical validation requires prospective, multi-center studies that assess AI performance in real-world settings. To date, only a minority of models have reached this stage. When external validation is performed, performance often drops substantially. Besides, studies rarely evaluate AI-assisted clinician performance or demonstrate improvements in patient-relevant outcomes, which is an important consideration for clinical adoption. The lack of interpretability which often referred to as the “black-box” problem, further undermines clinician trust, particularly for irreversible intraoperative decisions.

Regulatory authorization represents a critical realistic barrier. Unclear regulatory pathways and the gap between research prototypes and medical device requirements prevent most models from transitioning from publications to deployable tools. Liability attribution also remains unresolved. Surgeons bear ultimate responsibility but have no practical way to verify AI recommendations before acting on them, fostering caution and delaying adoption.

Post-deployment monitoring is another neglected issue in the AI-assisted application. Even after regulatory approval, models may experience performance deterioration over time as patient populations, imaging equipment, or clinical protocols evolve. These changes are often subtle and cumulative, making them difficult to detect without systematic surveillance. Continuous performance monitoring, cybersecurity, data governance, and clear accountability mechanisms for incorrect recommendations are rarely established before deployment, yet they are critical for maintaining safety and trust. Human-AI interaction also requires deliberate design, not only to ensure interpretability and clinical relevance, but also to avoid automation bias and alert fatigue, which can undermine rather than enhance clinical decision-making.

Beyond these stage-specific challenges, successful AI integration also depends on early attention to workflow integration, user-centered design, and ethics, including data privacy, informed consent, and algorithmic transparency. Rather than developing algorithms in isolation, AI tools should be conceived with clinician-led problem formulation from the outset. Workflow integration requires that AI outputs fit existing routines, and user-centered design ensures that tools are intuitive and clinically relevant. Ethical considerations must also be embedded from the start. Yet, clear policies for these issues remain underdeveloped in most systems.

AI should be positioned as an assistant rather than a replacement for surgical judgment, and trust develops when clinicians can understand and verify AI recommendations through active engagement rather than passive acceptance.

### 6.2 Limitations of this review

Several limitations should be acknowledged. First, as a narrative review, study selection and synthesis are inherently subjective, and relevant studies may have been omitted despite a structured search. Second, the included studies are heterogeneous in design or other characteristics and we did not perform formal quality or risk-of-bias assessment, precluding quantitative synthesis. Third, this review focuses on the surgical pathway and does not cover pathology, radiotherapy, or systemic therapy. We have also restricted our scope primarily to models specifically developed or validated for colorectal cancer, other models across gastrointestinal malignancies are not discussed in detail. Fourth, the rapid evolution of AI means that recent or unpublished studies may have been missed. Fifth, as with any review, our findings are time-bound and may require updating as new evidence emerges.

## 7 Prospective and outlook

AI has demonstrated substantial potential across the entire procedure of CRC surgical management, from diagnosis and intraoperative navigation to postoperative prognostication (**Figure 2**).

**Figure 2:**
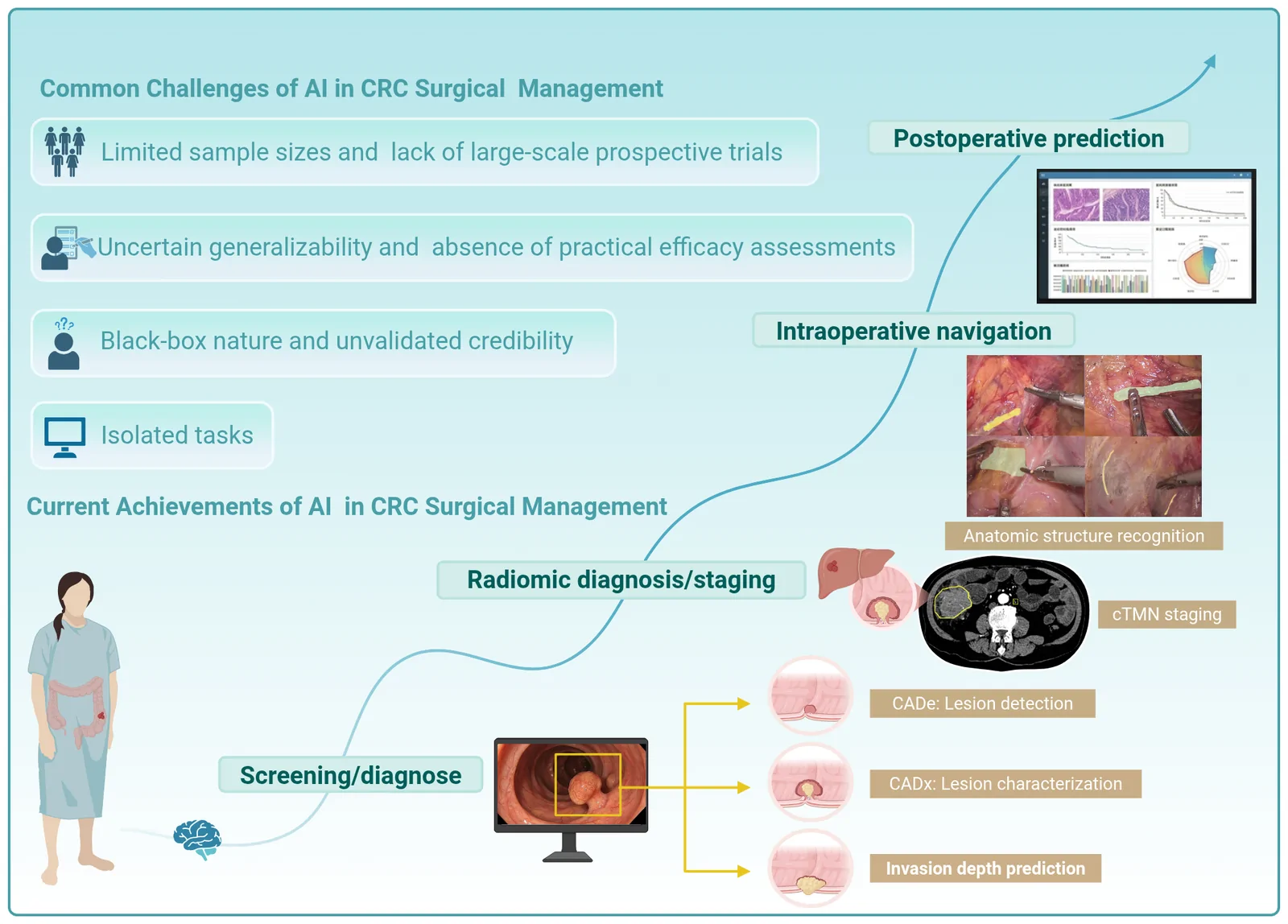
Current application of artificial intelligence in colorectal cancer management and challenges encountered. All clinical images were obtained from the authors’ institutional database and are used with permission.

However, a critical disconnect exists between the task-specific nature of current AI research and the integrated needs of clinical practice. The true value of AI lies in its seamless integration across the full CRC surgical treatment pathway, accelerating clinical workflows and potentially delivering patient benefits through earlier diagnosis, timely intervention, and evidence-based guidance. Current evidence supports technical feasibility and promising diagnostic or predictive performance in selected settings, whereas broad clinical effectiveness, safety, cost-effectiveness, generalizability, and patient-level outcome benefits remain insufficiently established.

From a surgical perspective, a key surgical need is proactive AI-based navigation that predicts anatomical variations and risk zones before they are encountered, enabling injury-avoidant dissection. Hepatobiliary surgery has made earlier progress in this direction with certified AI systems for preoperative 3D planning and surgical map generation (e.g. IQQA-Liver, FDA-cleared and CE-marked; InferOperate Suite, NMPA-approved and FDA-cleared), a disparity attributable to the quantifiable, anatomy-driven nature of liver surgery. Colorectal surgery, where outcomes rely more on nuanced intraoperative technique than static anatomy, must find its own path.

Translating AI into practice requires a clinically grounded roadmap. First, AI tools must be validated on patient populations that reflect the local caseload, ensuring their predictions are relevant to actual patients. Second, predictions must be delivered within the clinical decision window, with preoperative tools for surgical planning and postoperative models for adjuvant decisions. Outputs must be intuitive enough to be understood at a glance during busy clinical work. Third, AI should augment rather than disrupt surgical workflow. Seamless integration with existing systems and minimal cognitive load is essential for routine adoption. Fourth, surgeons must retain final decision-making authority, with AI serving as a second opinion rather than an autonomous advisor. Finally, the ultimate benchmark for any AI tool is whether it helps surgeons achieve better patient outcomes, including fewer complications, more appropriate chemotherapy use, and improved long-term survival, not whether it achieves higher statistical metrics. This surgeon-centered roadmap shifts the focus from technical optimization to clinical utility and patient benefit.

Future efforts should focus on interdisciplinary research, multimodal data integration, and surgeon-centered design to bridge the gap between technical promise and clinical reality.
